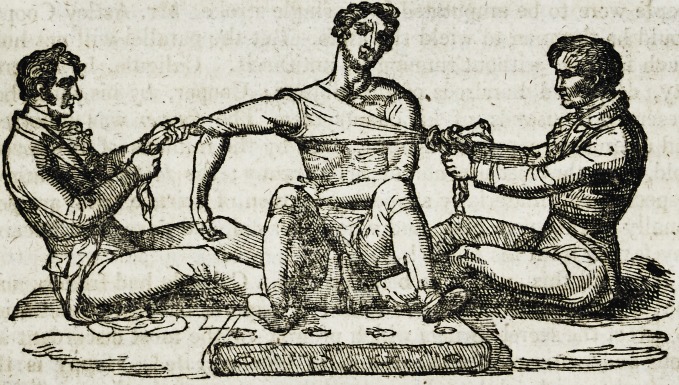# Transactions of Societies, &c.

**Published:** 1820-01-01

**Authors:** 


### Transactions of Societies, &c.

I. *Dublin Hospital Reports.* Vol. II.

II. *Transactions of the King's and Queen's College, Dublin.* Vol. II.

III. *New York Medical and Surgical Register.*

IV. *Surgical Essays.* By Messrs. ASTLEY COOPER and B. TRAVERS. Part II.

V. *Kirby's Cases in Surgery.*

VI. *Private Correspondence.*

IT is, no doubt, wisely ordered by Providence, that Societies can never act *collectively* with the multiplied energies and talents of the *individuals* composing them. If such were the case, solitary exertions would be annihilated, and monopolies in Science and Literature would operate with the same baneful influence which marks their steps in trade. In medical Science, indeed, there can hardly be any such thing as a *homogeneous combination* of exertion. The investigations of individuals can only be placed in apposition or opposition; and after all, it is by a single head that *principles* can be drawn from the congregated mass of materials thus collected by the most august societies. But the period for drawing these principles does not seem to have yet arrived, and therefore our business is, at present, to store up facts, as substrata for the construction of the future edifice.

### ART. 1. *An Account of an Endemic Disease of Ceylon, entitled* BERRI BERRI. By J. RIDLEY, Esq.

[ Dublin Hospital Reports, Vol. II. ]

THE term BERRI BERRI has been given to this fatal disease by the Malabar Physicians, signifying that waddling unsteady motion observed in sheep when walking, and which obtains in patients labouring under this disease. That restlessness, or panting for breath also, produced by the anxiety and distressing sensations then experienced, is expressed by the term BERRI BERRI.

Œdematous swellings of the legs and feet are among the first symptoms. The throat is frequently swollen, with a sense of numbness round the mouth; indeed, a general sense of numbness prevails, especially of the inferior extremities, which are unusually weighty and rigid; (hence the unsteady gait) with scanty, high-coloured, and acrid urine, ending, as the disease advances, in a total suppression of the secretion. A sensation of pain and tightness is felt immediately beneath the inferior edge of the sternum, which becomes so distressing as to induce the patient to solicit that the part may be cut open, under the hope of relief. The bowels are generally costive; the stomach irritable; the dyspnœa harrassing, when moving quick, or ascending an eminence; inability to lie down. The skin is natural until the advanced stage, when it becomes cold and clammy. The pulse is sometimes regular, sometimes quick, hard, or intermitting.

The approaches to this disease appear to be gradual, the patient generally perceiving more or less swelling in the legs, attended with lassitude, languor, and some dyspnœa, for two or three days prior to his applying for medical aid.

The appearances on dissection are not always the same. There is, however, in all cases, effusion of water into some of the cavities of the head, chest, or abdomen, independently of the anasarcous effusion. The heart, in some cases, is enlarged, with hydro-pericardium. The cellular substance of the lungs was found loaded with water, in many cases, by Dr. Christie. Mr. Ridley found the viscera inflamed, in the majority of instances; the diaphragm particularly so, as also the urinary bladder.

According to Dr. Christie, men of every constitution are occasionally attacked with Berri Berri; though the aged and debauched are more predisposed to it than others. Those also who have once had the complaint are most subject to it in future. Dr. C. found tailors, shoemakers, and other workmen, who lead sedentary lives, and spend much of their earnings in drink and debauchery, more frequently affected with this complaint than people of opposite characters.

The same intelligent physician informs us that he never met with an instance of the complaint in a woman, an officer, or a boy under twenty years of age. He also thinks, that a stay for some months on the station, is almost essential for the production of the disease; and that the greatest predisposition to it exists, when troops have been about eight or twelve months in the settlement.

*Treatment.* Dr. Christie's plan of treatment was a course of calomel and squills, the perspiration and other secretions being promoted by diluents and antimonials, while the strength was supported by cordial liquors, as gin, punch, &c.

Mr. Ridley's plan was very simple: a purgative of calomel, jalap, and crystals of tartar, was first given; the legs and feet were bathed in warm water, and afterwards well rubbed with camphor and oil of turpentine, or with mercurial liniment, and then rolled with a moderate degree of pressure, in flannel bandages. A pill composed of one or two grains of calomel, and two or three of powdered squills is then given every two or three hours, and the solution of crystals of tartar as common drink, sometimes made into punch, with Arrack or Geneva. Under this treatment, the disease is frequently removed in a few days. But when the complaint advances, it requires a more powerful practice, such as blisters to the back of the neck, and to the epigastrium; the warm bath; fomentations to the abdomen and lower extremities, followed by frictions of mercurial ointment, camphor, and oil of turpentine, while purgative glysters are to be thrown up, to allay the gastric irritability. When a paroxysm of vomiting or dyspnœa has been urgent, large doses of laudanum and brandy have been given with good effects; and in many desperate cases, these medicines have suspended those alarming symptoms, and time has been obtained for the employment of other remedies. Mr. R. administered a diuretic, composed of half an ounce of nitre and two ounces of distilled vinegar, and given in doses of half an ounce every three or four hours, with the best effects. Tinct. lyttæ was also employed with advantage. Gamboge was occasionally administered by Mr. Ridley, in small doses, either alone, or combined with the purgative powder.

It was found necessary, as a matter of HYGIENE, to have the men paraded twice a day, and then carefully examined, so that the disease might be detected in its primary movements. The natives were, by much persuasion, induced to sleep within doors, and drink a small quantity of Arrack, as a preventive of Berri Berri.

"Dissolution (says Mr. Ridley) took place generally in a sudden manner; very frequently while speaking to one man, I have been called to another, whom I had just left under promising circumstances, and have found him gasping, his eyes protruded, his hands clenched, and a few minutes have closed the scene. It has sometimes happened, that the man I was addressing has been taken off in the same manner."

Mr. Ridley, at length, became affected with the disease, and nearly fell a sacrifice to it. He recovered, however, by the plan of treatment already described.

The *Ætiology* of Berri Berri is but imperfectly known. Bad water, unwholesome diet, and certain states of the atmosphere, are generally considered as the principal causes, particularly the hot land winds.

### ART. 2. *Remarks on the supposed Effects of drinking cold Water; illustrated by Cases which occurred during the hot Weather of the Summer of* 1818. By JOHN WATTS, M. D.

[New York Medical and Surgical Register.]

IN the course of three days, in the summer of 1818, thirteen persons were admitted into the New York Hospital, with *apoplexy*, said to have been occasioned by imprudently drinking large quantities of cold water. They were principally common labourers, who had recently arrived in America. They had been exposed to the sun, while at work, all the morning; and were said to have drunk more than their usual quantity of ardent spirits. After dinner, they returned to work; and being thirsty, had gone frequently to the pumps, where they drank freely of cold water.

"The symptoms which occurred, were a sudden pain in the stomach, followed by slight vomiting, giddiness of the head, and fainting; shortly after, deep sighing, and difficult breathing; a rattling in the throat, with a secretion of a thick glairy mucus. The face was suffused with blood, and of a livid colour; the pulse was strong and full; the skin very hot and dry. In a short time after, coma supervened, and, in some instances, complete apoplexy, marked by stertorous breathing and loss of all voluntary motion; in most of them, tympanitis occurred to a very great degree." 82.

"The mode of treatment that seemed best adapted to this form of disease, was very large and repeated bleedings, stimulating enemata, and rubbing the whole surface of the body with aq. ammoniæ. This last application had a decidedly good effect in lessening the heat of the body and the comatose state of the patient; sometimes rousing him from an apparently fatal torpor. When the apoplectic symptoms had not yet supervened, laudanum, æther, and other stimulants, appeared to be beneficial, but their good effects were only momentary; for in a very short time afterwards, apoplectic symptoms occurred. Several of the patients were bled to sixty, and one of them to eighty ounces, in the space of two hours. The recovery of this last patient was more complete and rapid than any of the others." 83.

"As soon as the apoplectic symptoms were relieved by venesection, the patients craved for cold drink, and when indulged, vomiting often instantly succeeded; the effect of which was highly beneficial.

Two of the thirteen patients died; one of whom, on examination, presented the following appearances.

"Vessels of the brain extremely turgid; about five ounces of extravasated blood on the surface of the dura mater; some at the base of the brain, and the ventricles completely filled with serum. Lungs engorged, and apparently inflamed; heart red, and the pericardium much distended with a blood-looking fluid; stomach perfectly natural and quite empty."

The assemblage of symptoms here enumerated, Dr. Watts remarks, were widely different from those recorded by medical writers, as resulting from drinking cold water in a highly heated state of body. We have not the smallest doubt, indeed, of the complaint being *Coup de Soleil*, of which we have seen many instances. The drinking of cold water at the time, from the well known sympathy between the stomach and brain, might have helped to develope the disease, and be thus put down by the vulgar in the relation of *cause*. Much mischief would have ensued in these cases, (and in private practice did ensue) from administering laudanum,

æther, &c. the common remedies administered to counteract the effect of cold water drunk when the body is heated.

### Art. 3. *Case of Apoplexy, in which the fleshy Part of the Heart was converted into Fat.*

By J. Cheyne, M. D.

( Dublin Hospital Reports, Vol. II. )

A. B. 60 years of age, sanguine temperament, circular chest, and full habit, lived a sedentary life, indulging occasionally in the luxuries of the table. Having had some severe attacks of gout, he took magnesia for some years, which almost entirely suspended the regular paroxysms. To dry catarrh and œdema of the ankles he had been subject for two or three years, with an occasional intermission of the pulse. In January 1816, he consulted Dr. Cheyne for a pain in the right side, under the false ribs. He was prescribed opening medicines, and low regimen, both of which he neglected. In February, after an exhausting walk, he returned with fluttering or palpitation of the heart, which was relieved by six or seven glasses of wine. At nine in the evening, he was attacked with a violent fit of coughing, during which he fell from his chair, in a state of insensibility. In a few minutes he recovered sense; and Dr. Cheyne found his pulse extremely irregular and unequal; bounding quickly for several pulsations, and then pausing. Venesectio ad ℥xvj. The pulse continued irregular; the face became flushed; the suffocative cough returned; and he complained of pain in the occiput; fiat venesectio ad ℥xij, when the pulse became softer, though still irregular. Leeches to the head; colocynth and calomel pills. Next day he had several large bilious stools; his understanding was unimpaired; his recollection restored in a good measure. From this time he gradually emerged from the cerebral affection; but the cough, with pain about the region of the heart succeeded, followed by œdematous ankles; paucity of urine; great irregularity of action in the heart. The dropsical symptoms increased, and diuretics were prescribed. He was one morning unexpectedly found flushed, speechless, and hemiplegiac in his bed. All attempts to relieve him were unavailing.

*Dissection.* Serous effusion of three or four ounces in the ventricles of the brain, which was pale, but firm, with some marks of preceding inflammation of the meninges. About two ounces of fluid in the pericardium; heart three times its natural size; lower part of the right ventricle converted into a soft fatty substance; the upper part remarkably thin, and gradually degenerating into the morbid structure described. The cavity of the left ventricle greatly enlarged; the whole substance of the left ventricle, with the exception of the internal reticulated structure and carneæ columnæ converted into fat. Valves sound; aorta studded with steatomatous and earthy concretions. The liver was not examined; but Dr. Cheyne remarks,

"I am persuaded that diseases of the liver, which do not end in structural changes, often produce the greatest disturbance of the constitution, laying the foundation of fatal diseases of distant organs." p. 217.

Our Readers will not fail to perceive a striking similarity in the cardiac disease of this gentleman and that of Dr. Primrose Blair, detailed in the Fifth Number of this Journal. A somewhat similar case of the same is related in the Edinburgh Journal for 1816, by Dr. Duncan, Junior.

There is another curious coincidence in the cases of Dr. Cheyne's patient and Dr. Primrose Blair, namely, a pain in the *right* side of the thorax. As the various and anomalous pains, and other uneasy sensations in cardiac diseases have not, it is thought, been accounted for satisfactorily, on *neurological* principles, we shall here introduce some ingenious remarks on this subject by Dr. Foster, of Oakes, suggested by the perusal of Dr. Blair's case. Dr. Foster has long directed his attention to diseases of the heart, on which he wrote an excellent Thesis for graduation at Edinburgh,* and in a private letter expresses himself thus,—

"I am convinced, that the pain in the shoulder in *hepatitis*—that of the left arm in *angina pectoris*, and those various and hitherto unaccountable pains in different parts of the body, in *organic* affections of the *heart*, are produced in the following manner.

"The most prominent symptom in every case of both diseases is commonly turgescence and over-distension throughout the whole venous system. This is abundantly proved, in diseases of the heart, by almost every case on record. I have repeatedly seen the whole venous system gorged with blood. When we recollect the very intimate connexion which subsists between the nerves and the veins in their course; how, for example, the par vagum is bound up in the same sheath with the internal jugular vein, we shall be at no loss to account for sympathetic nervous pains. It is well known, that considerable pressure upon a nerve produces insensibility, while slighter pressure causes pain to be felt at the end of the nerve. Now, the pressure of the enlarged and congested venous trunks upon the accompanying nerves, appears to be the true cause of the pain in the shoulder in *hepatitis;* and the pain in the thorax and arm, in diseases of the heart. This at once shew us why the pain was seated in the right side in Dr. Blair's case, viz. because, in that side the two *venæ cavæ* and the large venous trunks which terminate in them are situated, which produced a greater congestion on that, than on the left side. Every one who recollects the intimate union which exists between the subclavian vein and the great nerves of the axillary plexus, will be at no loss to see how pressure should produce a pain in the extremities of those nerves

* This Thesis we have read, and shall probably introduce some extracts from it on a future occasion.

of the arm. The nature of the sensation felt too, is similar; the pain felt in the little finger, when the elbow is struck and the ulnar nervepressed upon, is not dissimilar to the dull aching pain of *hepatitis*, although more severe and transitory. By this explanation, we may farther account for sympathetic pains which are unaccountable on any other hypothesis. In some of Dr. Ferriar's cases, the sympathetic pain was felt in the abdomen. How shall we account for this by pursuing nervous ramifications? But the turgescence extended throughout the whole venous system, yet probably being the greatest in the abdomen, the nerves there were more pressed upon, and pain induced.

"One of the following symptoms commonly attends and indicates speedy dissolution in diseases of the heart, viz. rupture of vessels, effusion of water, or effusion of blood. It is worthy of remark, how very much the degree of tone or of laxity in the circulating system modifies these diseases. In some very rare instances, the vessels are so firm and rigid, that no effusion whatever occurs, and here the patient suffers repeated exacerbations of difficult breathing, and the constitution bears up long against the disease. In other cases, the vessels are more relaxed, and dropsy follows. This is the most common termination of disease of the heart. Where they are still more relaxed, blood is extravasated, and in these cases the patient goes off suddenly. But in general, in organic affections of the heart, the patient does not die in a fit of syncope. In the case of Dr. Blair, it is obvious, that the bursting of the aneurism was the immediate cause of death."

*Private Correspondence.*

As an interesting Sequel to the above, we shall here introduce a valuable Case transmitted to us by Mr. George Williams, Surgeon, of Portsea.

"John Denham, aged 38 years, of regular habits, applied for medical assistance on account of a difficulty of breathing, with which he had been affected for four months. Ten months since, he got wet through, and neglected to change his clothes for some hours; which imprudence was followed by a violent attack of acute rheumatism; on the subsidence of which, the affection of the chest, now complained of, first made its appearance, and has become gradually and progressively worse.

"At first sight, I was struck with the peculiarity of his breathing and the anxiety pourtrayed in his countenance, and felt a conviction they were indicative of some serious thoracic disease; more minute examination too firmly established this.

"The respiration was quickened by the least exertion, accompanied with a hissing sound, like a cnrrent of air rushing through a dry tube. During each inspiration the head was thrown back, the eye-brows elevated, the mouth opened, and the nostrils expanded, as it were, eager to catch every breath of air. The moment he attempted to lie down, a teazing, dry, cough obliged him to resume the erect position, or to raise the trunk and head by means of pillows. It was seldom he could obtain a moment's sleep, without being awoke with horror from frightful dreams, and seized with a violent palpitation and 'death-like' sensation at the heart.

"The pulse was 120, thread-like, but regular and without intermission. On applying the hand to the region of the heart, the action of that organ was obscure and scarcely to be felt. Thoracic percussion elicited a dull, obtuse sound; abdominal pressure caused a distressing sense of suffocation, and acute pain under the centre of the sternum.

"The bowels executed their office correctly, and from the appearance of the fæcal discharges, the function of the liver was undisturbed. There was tenderness, extending through to the spine, on pressure at the præcordia.

"The urine was scanty, and deposited a copious lateritious sediment. No œdema of the legs; no pain, except about the throat and between the scapulæ, but this not violent nor constant. Insatiable thirst; white chalky tongue; dry, parched, and heated surface with frequent flushings, and pallor of the face.

"Under these circumstances, it was thought advisable to abstract some blood; as the first cup-full exhibited a highly inflammatory appearance, another and another were taken, with evident relief to the pulse and respiration. A large blister was applied to the chest, and he took a combination of squill and calomel, with the infusion of digitalis. He was directed to raise the upper posts of his bed, by inserting two bricks beneath them; to drink plentifully of cream of tartar tea; to keep his mind as tranquil as possible, and to avoid any violent or sudden exertion.

"After this plan had been continued for a week, with a repetition of the bleeding, an evident improvement had taken place in the state of the patient; the respiration was freer, the skin cooler, the urine augmented.

"From the fear, as I afterwards learnt, of incurring greater expences than his finances would permit, I saw or heard no more of my patient till the expiration of six weeks, when I was sent for, at his request, 'to witness his last moments.'

"I found him sitting upright in bed with a pallid, most anxious, and emaciated countenance; his lips livid; his respiration hurried in a violent manner; pulse scarcely to be felt, much less numbered. He complained of a stinging pain down the arms; just over the pubis, and all around the rim of the belly. Tenesmus, a frequent attendant on disorder in the respiratory apparatus, as pointed out by Dr. Bree, was a very distressing symptom.

"The action of the heart, hardly to be felt in the chest, and but slightly at the scorbiculus cordis, was tremulous and rapid. The legs œdematous; the hands swoln, and the extremities cold. He lived three days in this state. A constant dread of going to sleep, from a conviction that he should never awake again, harassed the last few days of his life.

" *Dissection.* The lungs sound; the right lobe adhering very closely to the pleura costalis; no effusion in the cavity of the pleura. The pericardium contained eight ounces and a half of reddish serum. The heart occupied nearly the whole of the left cavity of the chest, (compressing the lung on that side so as must have materially impeded the free execution of its function) and intruding considerably into the right; it was full thrice its natural size, soft and flaccid in structure. The parietes of the ventricles much augmented in thickness. The reflected surface of the pericardium which envelopes the heart, was here and there studded with white points, raised above the surface, apparently coagulable lymph; and at the apex, a number of little sanguineous effusions resembling petechiæ, were observed between this membrane and the muscular substance of the organ.

" That part of the arch of the aorta before it gives off the large vessels, bore evident marks of the sequelæ of inflammatory action; the whole of its coats being irregularly thickened in some places, and much attenuated in others; its internal surface had lost its natural polish, was rough and thickened by the deposition of a white cheesey substance; its calibre, for the space of two inches from its origin, increased one-half.

" The viscera of the abdomen were all healthy in appearance; the liver rather larger than usual.

" From the occurrence of the thoracic affection, immediately on the subsidence of acute rheumatism, one is inclined to suspect some metastasis of morbid action, and that, most probably, the organic disease we are here presented with, had its origin in rheumatic inflammation; a cause of cardiac disease, alas! too frequent.

" The analogy between this poor fellow's sufferings, and those of a patient labouring under spasmodic asthma, was very great, but there was more distress about him; more anxiety; more difficulty in laying down; more disturbance from unpleasant dreams. Besides, all these symptoms were constant, there was no intermission. If I mistake not, asthmatic patients in general, bow the head forwards and raise the shoulders, to facilitate inspiration; here the head was thrown back; the muscles attached to the sternum, not those connected with the scapulæ, were put into action.

" The peculiar working of the nostrils, which is often observed in pulmonic affections was, in this instance, very remarkable. Am I deceived, or does this not furnish a diagnosis in those affections of the lungs so common in children? Is not this working of the alæ present when the lungs are actually inflamed, and absent in those spasmodically, or rather sympathetically, affected from stomachic or other irritation?

" Soreness and pain round the rim of the belly is almost universally complained of by patients labouring under structural or functional disease of the central organ of circulation; in the instance just related, this was a prominent symptom. How is it to be accounted for?

"On perusing the records of medicine, one is struck with the apparent increase of cardiac affections which has taken place within the last few years. Is this not to be accounted for by adverting to the spirit of inquiry; the independence of scholastic dogmas; the more intimate acquaintance with morbid anatomy, and the phenomena of diseases connected with it, which characterize the medical practitioner of the present day, rather than to any actual increase of the diseases in question?

"No doubt many diseases, which have been classed and confounded under the general terms asthma, nervous complaints, dropsy, &c. by our professional brethren of former days, had their seat and origin in disorder or diseases of the heart.

"The influence of the mind over the functions of the heart is well known to the physiologist, and indeed to every one; and, probably, the increased sensibility of the former, from its more general expansion and cultivation in this age, when all are learned, may, in some measure, tend to the more frequent production of diseases in the latter."

### Art. 4. *An Account of an uncommon Disease of the Hand and Fingers.* By C. H. Todd, Member of the Royal College of Surgeons.

[ Dublin Hospital Reports, Vol. II. ]

This is an affection bearing a resemblance, in some points, to the cutaneous whitlow, and is designated by Mr. Todd, the "*Paronychia cutanea maligna.*" He looks upon it as being of more than local character, and as connected with a morbid state of the digestive organs, or of the system at large. It is preceded by symptoms indicative of debility, and want of energy in the functions of assimilation; such as inappetency, flatulence, thirst, irregularity of the bowels, depression of spirits, &c. Then the local affection takes place, and is most commonly felt in the night, for the first time, the patient being annoyed by stinging pains in the fingers or hand. At this period, small red or livid spots, without hardness or elevation, may be observed, which soon become black. The sensation in the part is between soreness and itching, and the patient rubs or scratches it. This accelerates vesication, the cuticle becomes detached, and a thin offensive sanies is effused under it. When the vesicles are removed, the subjacent skin appears sphacelated, and superficial ulcers are discovered, the disease shewing a disposition to extend by destroying the surface merely. Local pain, at first, is severe; but subsides in a few days, which is a bad sign.

During the whole progress of this disease, the patient labours under a low fever; sometimes assuming the typhoid type. In every instance the functions of the stomach are impaired, and the secretions diminished, with mental depression and anxiety.

The indications of treatment are, to counteract the constitutional disease and debility, and to improve the condition of the digestive organs. The local applications most useful were spirituous fomentations, or camphorated embrocations. When the vesicles form, all the diseased cuticle ought to be cut off with the scissars as early as possible, and warm terebinthinate dressings applied.

### Art. 5. *Case of long-continued Pyloric Ulceration, with the Appearances on Dissection.* By William Brigham, Surgeon, Manchester.

John Hatton, ætat. 40, by trade a machine-ruler, had been visited by me, at different periods, during the last three years of his life. He dated the commencement of his complaint fifteen years back.

The following are the prominent features of his malady, partly taken from his own account, partly from my own observations during the time of my attendance. Extreme emaciation; countenance indicative of great bodily suffering; skin yellow and dingy; position always half-bent; temper very irritable, and passionate, especially when under a paroxysm of pain. He had, when twenty years of age, been subject to difficult digestion, and occasional pains in the epigastric region, with frequent vomitings after taking food. These symptoms were succeeded by more dull and deep-seated pains in the same place, generally increased after eating, together with a hard, irregular tumour there, which was painful on pressure. The tongue was now always dry, and covered with a white thick fur; the pulse generally slow, weak, and regular; urine scanty; bowels habitually costive; often ten days without a motion, though taking the most drastic purgatives, consisting of calomel, scammony, and gamboge. During the last fifteen years, the pains in the stomach were generally violent till relieved by the vomiting of his food, when he would return to eating with great voraciousness.

He suffered much from colicky and flatulent pains in the stomach and intestines. The matters vomited generally appeared, on minute examination, to consist chiefly of mucus, with a thick cream-coloured substance, of tenacious feel, and peculiarly sour smell, resembling fresh barm. He would frequently throw up six or seven pints of this mucous fluid in the course of the day, either alone, or mixed with food. Such was the unvarying appearance of the ejected matters during the whole course of the disease.

When the hand was placed on the epigastrium, and pressed with tolerable firmness, it caused little or no pain; but when a single finger was pressed there, considerable pain and even disposition to vomit was induced. The eating of cheese always caused great uneasiness; and dried currants or pease would be thrown off by vomiting a fortnight after being eaten.

The foregoing symptoms continued, with little variation, for nearly *twelve years*, excepting occasional intervals of ease, and remissions of vomiting. After this period, the system began to give way; the complexion became quite yellow; the emaciation extreme; the vomitings very frequent; but the matters ejected still the same, and never presenting any brown, chocolate, or blackish hue, excepting once, about a year before his death, when the patient vomited a considerable quantity of a reddish coagulated substance, resembling what is observed in some cases of hæmatemesis. The patient at length, after a series of protracted sufferings, during which time all the above enumerated symptoms were more and more aggravated, became exhausted, and died.

*Dissection.* Assisted by my intelligent friend, Mr. Jordan, surgeon, whose zeal and anatomical skill have often afforded me much satisfaction in morbid investigations, I examined the body, twenty-four hours after death.

The colon, which was large and much distended, firmly adhered to the inferior edge of the stomach, and also to the gall-bladder. In passing along the right hypochondriac region, it made a sudden turn downward, and then took the usual course. The veins of the stomach and intestines were unusually large and congested; but the intestines themselves were free from disease. The stomach was much enlarged, and its coats, particularly about the pylorus, were remarkably thickened. It contained a considerable quantity of food, mixed with some dark-coloured mucus. The whole internal surface of this organ was very blood-shot, being covered with minute ramifications of distended blood-vessels, shooting from a central point, and giving a stellated appearance to the whole mucous membrane, especially towards the pylorus. The different coats of the stomach were strong, thick, and well-marked; the mucous tissue having a rich velvet appearance. The cardiac orifice was of the natural size, and structure; but the pyloric orifice was much contracted, the opening into the duodenum being not more than one-third its natural size; the structure round the pylorus was thickened. A defined oblong ulcer, four inches in circumference, almost surrounded the pylorus, having an irregular elevated edge, and deep hollowed base; in some parts firm and opake; in others, very thin, tender, and quite transparent. This ulcerated surface was covered by a thick mucus, of rather a dark colour. The other abdominal viscera were sound.

The foregoing case is remarkable, and also instructive, in shewing us the great length of time which the human frame will sometimes bear ulceration of the stomach. It shews us too, that dark, or chocolate-coloured vomitings are not invariable attendants on gastric ulceration of the scirrhus kind.

M. Bourdon, has lately read a Memoir in a medical society of Paris, where a case is detailed of scirrhus implicating the pylorus and whole body of the stomach, with the exception of the cardia, and yet no *vomiting* took place at any period of the disease. Symptoms of phthisis supervened, and a purulent fluid was found in the chest after death.

In a late Number of the Edinburgh Journal, Mr. Paxton relates the case of a shoemaker, who, for three years, was affected with pain and irritability of the stomach, so that solid food could seldom be taken without violent retching, and vomiting. One evening, being affected with intense pain in the epigastrium, he took two active emetics, but without effect. Pain and tension increased, extending to the whole abdomen. He died. The stomach was mortified towards the lesser curvature and near the cardiac orifice, and had given way to a considerable extent, whence had issued into the abdomen three or four quarts of a dark-coloured fluid. The pylorus was scirrhous, and the orifice contracted.

### ART. 6.—*Case of Suppuration in the Liver.* By JOHN WATTS, M. D. of New York.

[ New York Medical and Surgical Register. ]

THE following Case deserves very attentive perusal. We shall condense it, but in the author's own language.—B. B. ætat. 30, stated, that in the summer of 1816, when employed as a boatman, on a canal in South Carolina, he had an attack of ague which continued until the following April. Immediately after the fever left him, his feet began to swell, with pain in the region of the liver. He soon became completely anasarcous. He experienced some relief from bleeding, blisters, cathartics, and diuretics. He was admitted into the New York hospital on the 8th of January, 1818. His face was sallow and bloated; there was evident fluctuation of the abdomen; fulness and pain of the right side; the pain extending to the shoulder, and accompanied by a dry cough; pulse slow and feeble; respiration oppressed; bowels costive. A cathartic being premised, he was put upon the use of calomel and squills, with a blister to the side. Under this plan, a slight ptyalism was raised; his dropsical symptoms subsided, but he continued to have pain in the side and shoulder, with dry cough.

Early in February he caught cold, and the cough and pain became aggravated; but the pulse being small and slow, he was not bled, while the use of calomel was resumed, with a blister to the side. He continued to decline; and in ten days, an abscess of the liver burst, the matter being discharged very copiously from the mouth. He expectorated, for a long time, a quantity of purulent and bilious-looking matter, amounting to at least a pint a day. A generous diet and porter were ordered, with the view of supporting his strength under this copious discharge. The pain in his side increased; the cough was troublesome; his pulse became full and strong; and in twenty days from the bursting of the abscess, he was considered completely hectic, having a distinct chill and fever every night, succeeded by a profuse perspiration towards the morning. At this time he came under the Reporter's care. The tonic plan was continued about a week longer; yet it was evident that he grew worse; the expectoration became more profuse than ever, greatly exceeding a pint in the twenty-four hours, and a very high degree of excitement was observed after each chill.

The tonic and stimulating plan was therefore laid aside on the 9th of March, and the patient restricted to toast and barley water. Next day he felt better; his fever less violent; his expectoration diminished. A seton inserted in the side. His appetite being craving, small doses of tartarised antimony were ordered to excite nausea. He was much benefited by this mode of treatment; but complaining, a few days afterwards, of pains in his limbs, resembling rheumatism, he was bled to eight ounces, when he found that he could breathe much freer and easier. That evening he had no chill, and very little fever in the night. The expectoration was much less, as well as the cough; blood buffy and cupped. Antimony and digitalis ter in die.

On the 18th. Symptoms again authorised venesection, which he bore well, ad ℥xvj. His expectoration now became quite moderate, and he appeared in every respect much better. He complained only of a tendency to perspiration, for which he was ordered the diluted sulphuric acid. From the 20th of March to the 1st of May, he was bled seventeen times, averaging at least fourteen ounces each time. On one occasion, he lost nearly thirty ounces at once. During the whole of this period he was restricted to as simple and light diet as possible. The tincture of digitalis was given for three weeks, and gradually increased to one hundred and fifty drops per diem. Under this plan his hectic symptoms disappeared altogether, and he left the hospital for the country, with every indication of returning health. P. 140, 1, 2.

We are persuaded that the parietes of an internal, as well as of an external abscess, often continue a long time in a state of inflammatory congestion, which, under stimulating regimen, augments instead of diminishing the purulent discharge, with all its concomitants, fever, emaciation, &c. and we have had several opportunities of seeing the superior good effects in such cases of low regimen, perfect quietude, and occasional evacuations. The foregoing case and these hints will be worth attending to.

### Art. 7. *Case of Fracture of the Pelvis.* By Gideon Mantell, Esq. F. L. S.

Francis Pursell was admitted into the Royal Ordnance Hospital at Ringmer, on the 1st of September 1818. He had sustained a severe injury of the pelvis, from the horse on which he was riding having reared and fallen upon him. He complained of acute pain across the lumbar region, and through the pelvis; but, upon the most attentive examination, no fracture of the ossa innominata could be detected. He had lost the power of moving the lower extremities, but permitted me to bend and extend them, without expressing much uneasiness. The left thigh was shorter than the right, but the knee and foot were not turned from their natural direction.

A few minutes after his admission, above two pints of blood flowed from the urethra, and afterwards a small quantity of urine. The state of his pulse, and the appearance of his countenance, manifested a remarkable depression of strength; and it seemed probable that he could survive the accident but a few hours. The next day, however, his pulse had increased in strength and frequency, and he suffered greatly from retention of urine: he was bled, and immersed in the warm bath, but experiencing no relief, a catheter was introduced, and a large quantity of dark-coloured urine was drawn off. At the expiration of a week, the bladder had recovered its powers, and the use of the catheter was discontinued.

On the 12th of September, he complained of a swelling and tenderness on the inside of the left thigh, near the ramus of the pubis; the part was inflamed, and in the course of a few days, an evident fluctuation was perceptible. Ulceration speedily ensued, the abscess burst, and discharged nearly three pints of pus, urine, and fæces. As the opening was small, it was freely enlarged with a bistoury, which afforded the patient considerable relief; the urine flowed freely through the aperture in the thigh, and the quantity of pus discharged in the course of twenty-four hours usually exceeded two pints; pressure on the abdomen assisted its expulsion.

After the expiration of two months, the state of the abscess was but little altered: the patient had become hectic, and was much emaciated. By a liberal allowance of wine, and cinchona, with mineral acids, the symptoms gradually assumed a more favourable appearance; the discharge, though considerable, evidently diminished; the urine flowed through the urethra, and the shooting pains through the pelvis, of which he had continually complained, were diminished in frequency and severity.

From the latter end of December, his recovery was progressive; with the assistance of crutches he was able to walk across the ward.

In February 1819, the abscess was healed, the patient's health re-established, and he could walk with a stick, without experiencing much inconvenience. The shortening of the left thigh, however, rendered it necessary to send him to Woolwich, that he might be discharged.

Although, at the admission of Pursell into the hospital, there was no evident *crepitus*, nor displacement of the ossa innominata; yet, it appears to me, that the shortening of the left thigh, and the other remarkable features of the case, were occasioned by a fracture of the left os innominatum, (which probably extended into the acetabulum) producing injury of the pelvic viscera, and the formation of an abscess, which communicated with the bladder and rectum.

*Private Correspondence.*

### Art. 8.—*Acute Rheumatism, treated solely by Leeching.*

The following Note, made by Dr. James Clark, of Rome, in going round the Val de Grace Hospital lately, with M. Broussais, was kindly given to us by this intelligent physician.

"On going round the wards, a soldier was pointed out to me who had come into the hospital with severe acute rheumatism of both knee and ankle joints, accompanied with the usual symptoms of swelling and redness, &c.; fifteen leeches were applied to each joint. Next day he was free from pain, and the tumefaction of the joints was gone. Both thumb joints were soon after attacked, and several leeches were applied to them, completely removing the pain and inflammation. I examined his knees; the joints appeared perfectly natural. He heard the history of his case stated, and assented to the correctness of each particular. In this way acute rheumatism is treated at the Val de Grace, and with uncommon success. Gout is also treated in the same manner."

### ART. 9. *Remarks on Mr. D. Johnson's Paper on Hydrophobia.* By RUSTICUS.*

In the fourth Number of the Medico-Chirurgical Journal, Mr. D. Johnson has published an interesting account of a successful prophylactic treatment of hydrophobia. Fearing that some of your readers may suppose there is a difference between the disease in India and in Europe, and an impropriety in adopting the same practice in both countries, I take the liberty of referring them to the Nosologia Methodica of the celebrated Franc. Boissier de Sauvages, who practised in Germany, and in the year 1747 made this method of treatment the subject of a Prize Dissertation. As this work may not be in the hands of all your readers, nor perhaps of Mr. D. Johnson himself, I will transcribe a few passages.

" Multiplici observatione a viginti retrò annis constat, homines a *rabidis demorsos*, maturo et constanti *hydrargyri* usu ab *hydrophobia* antè, quàm accedat, immunes evadere; hoc docent observata clariss: *Desault Burdigalensis*. *Darluc Foroliviensis*, *Fratris du Choisel*, *Jesuitæ*, qui in Pondichery *trecentos* hoc methodo servavit; mea demum *pluries* a quatuordecim annis repetita; cum antè hocce inventum neminem vere hoc veneno infestum convaluisse vulgo creditur.

Ex diversis hydrargyri præparatis, forma turbith, cinnabaris, cet. præstantissima est *pomata vulgaris Neapolitana*,† cujus drachma quantocyus vulneri et viciniæ est inungenda; cino si tempus urgeat primâ vice semiuncia, dein singulis diebus ægro, e balneo exeunti, iteranda est inunctio unius, alteriusve tantum drachmæ, quod repetatur per quindecim, vel viginti, dies, ita, ut æger ad summum pauca sputa, plura quam pro solito more ejiciat; ea vero servatis servandis, sub victu refrigerante et humectante, vitando äerem frigidum, *de io huic morbo præcavendo* sufficiunt." Vide, *F. B. de Sauvages Nosolog. Method.* a C. F. Daniel, Lipsiæ, 1796. Tom. iv. p. 358 et seq.

Mr. D. Johnson is anxious that the term hydrophobia may be exchanged for one more correct. This has been done by Mr. John Mason Good, in his classical work on Nosology; and I hope to Mr. Johnson's satisfaction. In the Note on the Genus Lyssa, which is

---

* Rusticus is a Medical Practitioner of great talents in the West of England.—EDITOR.

† The pomata vulg. neapol. is, I believe, the common mercurial ointment.

derived from *Λύσσα*, rabies, Mr. J. will find that water-dread is not considered a pathognomonic symptom.

It is to be feared, that injustice has been done to the remedy in question, by the injudicious use of it as a *curative* instead of a *preventive* mean. The subject deserves farther investigation.

### Art. 10. *On Hydrophthalmia.* By Rusticus.

In addition to Dr. Kennedy's cases of this disease, contained in the fifth Number of the Med. Chir. Journal, I beg to inform your readers, that the practice he recommends was adopted by me, and proved successful, in a case which came under my care in the year 1814. The particulars would have been communicated before, had I not waited in hope of obtaining more experience in the disease, which fortunately is very rare. I am glad the subject has been taken up so ably, and feel pleasure in confirming Dr. K's observations.

In the case to which I allude, the eye was twice its natural size; and projecting through the eye-lids, rendered them perfectly useless. The disease had been advancing during several months, attended with violent pain in the forehead and temple. The patient was a lady, aged 70. The cornea was transparent, but the sight was lost *pro tempore*. One of the branches of the temporal artery was opened twice. After the second operation, the effusion ceased, and absorption proceeded rapidly; so that in twenty-seven days from the time I was consulted, the disease was completely removed, and the sight restored. External applications were of no avail; but I think the recovery was expedited by mercurial purges. The patient is still alive, and has her sight perfect.

If we may judge from the severe treatment adopted for the removal of hydrophthalmia, it must appear, until lately, to have been very imperfectly understood, in common with other serous effusions. It was the practice of St. Ives to extirpate the eye; and Scarpa, who has written on the principal diseases of the eye, advises the evacuation of the fluid, and the production of the suppurating inflammation, in order to insure the entire collapsion of the tunics, and the consequent destruction of the organ. For the same purpose, Mr. Ford, surgeon to the Westminster Dispensary, conveyed a seton through the distended globe; but in the cases related by him, it must be observed, that an opacity in the transparent cornea had previously destroyed the sight. See *London Medical Journal*, Vol. I. and *Medical Communications*, Vol. I. p. 409.

### Art. 11. *On Hydrops Ovarii.* By Rusticus.

This was formerly considered one of the opprobria of our art. The symptoms denoting its early formation were overlooked, and after fluctuation became evident, instead of attending to the relatively increased action of the arterial system, we were directed to attempt the absorption of fluid, which was supposed to have accumulated through a debility or torpor in the absorbents of the part; and if this did not succeed, our only resource consisted in the operation of paracentesis. Dr. Parry having established, and I trust the profession having by this time admitted the fact of a relative increased momentum of blood in part of the body, while the general circulation remains unaffected; it is easy to account for the structural derangements which may happen, while the pulse at the wrist may not only be slower, but smaller than natural. Viewing dropsy then as an inflammatory disease, it is rational to expect a cure from such means as may control the local increased action in, and subdue the morbid determination of blood towards, the affected region. One of the principal means of effecting these desiderata is bleeding, either local or general, according to circumstances; and Drs. Abercrombie, Kennedy, and Crampton, have rendered the profession a service by publishing this practice, and their sentiments on this subject. Not recollecting to have read in the modern publications any remarks on the utility of bleeding in the commencement of ovarian dropsy, it may not be uninteresting to observe, that in my own practice, this plan of treatment has been successful in three cases. The first patient was an unmarried woman, aged 35, who had observed an enlargement of the abdomen on one side for two years; and when I was first consulted, a distinct fluctuation was to be perceived. She had some tenderness and much pain. Leeches were repeatedly applied, and one blister; and at the end of three months the fluctuation was no longer evident, she became easy, and was able to follow her employment. This took place four years ago; and since that period she has experienced no relapse. Some fulness remains in the iliac region.

A lady, aged 20, unmarried, was the subject of the second case. She had observed an occasional fulness on the right side of the abdomen frequently, during six months; and when I saw her, the swelling had been permanent during several weeks. The ovarian tumour was as large as a child's head, tense, painful, and tender; and was more circumscribed than in the first case. The bowels were costive. Twelve or fifteen leeches were applied frequently; their repetition being determined by increase of pain and tenderness. A few blisters were also employed. At the end of twelve months the tumour and other symptoms were nearly removed; and she has remained well the last two years. Some enlargement of the ovary is still perceptible, and will probably continue.

The last instance occurred in a married woman, mother of several children. She had advanced in life further than the others, but had discovered the tumours only three months, during which time they had rapidly increased. A slight fluctuation, but no pain or tenderness was manifest. She was bled in the arm, and blistered, and local bleeding was repeated three times. At the end of six weeks, the fluctuation disappeared, and the tumours became stationary. Both the ovaria were diseased, and several of her relatives had died of ovarian dropsy.

In all these cases, due attention was paid to the stomach and bowels; but *no diuretics* were administered.

Art. 12. *Of unnatural Apertures in the Urethra, and on encysted Tumours.* By Astley Cooper, Esq.*

The volume from which we have selected the present paper for analysis, exhibits a striking illustration of the observation with which we set out in this article; namely, that "societies can never be expected to act with the combined energies of the individuals composing them." The first part of these Essays, the work of only two individuals, has passed through *three editions*, in a very short space of time! We need not make any *comparisons* to elucidate the object of our remark. We have heard objections made, indeed, to some articles in the first part of these Essays, by men of eminence, particularly to the papers on iritis, and on affections of the prepuce; but, upon the whole, few works have been received by the profession with such universal and flattering marks of approbation as the Essays under review.

The second volume, or part, is entirely Mr. Cooper's; and although, from the nature of the subjects embraced, we conceive it to be not more generally interesting than its predecessor, yet it abounds with exceedingly important matter, and is worthy of the high profesional source whence it emanates. The work contains three Essays—the first, occupying the greater portion of the volume, is on various dislocations and fractures of the lower extremity, of which we shall give a full account in the next number of this Journal. In the mean time, we shall here present our readers with an analysis of the second and third Essays, on the urethra, and on certain encysted tumours.

I. Artificial openings into the urethra, made in surgical operations, generally heal without difficulty; but it is not so when apertures in this canal are the result of diseased states of the constitution or of the urethra. Fistulous openings are most commonly the consequences of stricture. The obstruction to the flow of urine, and the pressure of this fluid on the sides of the urethra, behind the stricture, lead to ulceration. The irritation of the urine next causes inflammation and abscess; and, when the matter is discharged by nature or art, the water follows its course, and usually continues to do so, till the stricture is removed. It follows from this, that these abscesses, the precursors of fistulæ, should be opened as soon as it is ascertained that they contain matter. This promptitude is advantageous in preventing extensive destruction of the parts by ulceration; and it not unfrequently happens, that the wound heals kindly, without any of those dangerous extravasations of urine into the scrotum, which often prove destructive to life.

The treatment of these fistulous apertures, when once formed, is sufficiently obvious. The strictured part of the urethra must be

* Surgical Essays, by Astley Cooper, F. R. S.; and Benjamin Travers, F. R. S. Part II. Octavo, 240 pages, and 8 plates. London, 1819.

widened by "the introduction of metallic bougies, increasing their size gradually until it reaches somewhat beyond that of the natural diameter of the passage." Sometimes it is necessary to introduce a large sized pewter catheter into the bladder, and there to let it remain, so as to conduct off the water; and, at the same time, extend the stricture and prevent the passage of urine into the false opening. Mr. Cooper observes, that caustic, which was formerly much employed, "is now comparatively seldom used;" yet, cases do occur where, from long neglect, the urethra and the parts surrounding the stricture become so disorganized, that no instrument can be passed through without an unsafe degree of violence. "Here the slower influence of the caustic will be attended with less danger than the use of the metallic bougie."

*Case* 1. A gentleman, after an abscess on the anterior and lateral part of the rectum, perceived that urine came along with the discharge. Mr. C. examined the urethra, and found some obstruction at the apex of the prostate gland. He advised the large metallic bougies; but a perseverance of several weeks produced no apparent advantage; the urine still passed by the fistulous aperture in the rectum. The metallic catheter was next tried, and the urine conducted through it for a month; but as soon as the instrument was withdrawn, the urine resumed its former unnatural course. The following operation was then performed.

"I introduced a catheter into the bladder, and my finger into the rectum, and then made an incision, as in the operation for the stone, in the left side of the rapha, until I felt the staff through the bulb. I then directed a double-edged knife across the perinæum, between the prostate gland and rectum, intending thus to divide the fistulous communication between the urethra and the bowels. A piece of lint was introduced into the wound, and a poultice was applied over it. When the lint was removed, the urine was found to take its course through the opening in perineo; the aperture in the rectum gradually healed, and that of the perineum quickly closed; after which, the urine took entirely its natural course." 200.

People of broken or naturally feeble constitution, will have a slight discharge from the urethra, without any previous disease or gonorrheal infection. A swelling forms in a line with some part of the urethra, and suppurates. The urine follows the course through which the matter flows externally, and a considerable discharge still continues from the urethra. "These (thinks Mr. Cooper) arise either from ulceration in the mucous membrane of the urethra, or from abscesses in the lacunæ; and, I believe, more frequently from the latter than the former. It is the usual practice in these cases to begin directly with the use of the bougies, but it is not judicious to do so, as they only add to the tendency to ulcerate, and increase both the local and constitutional irritation." 201.

We have so frequently experienced the truth of these and the succeeding observations, that we felt great pleasure in finding our sentiments confirmed by such authority as that of Mr. Cooper.

*Case* 2. A nobleman came to London with one of the abscesses above described, and with a copious discharge from the urethra, his constitution being enfeebled, and having suffered much from local irritation. A bougie was once passed into the bladder, but no stricture was discovered. The abscess was poulticed, and the matter discharged by the perineum, but still it continued to pass off both by the aperture and the urethra. By alterative and tonic medicines, the constitution was recruited, and the local complaint entirely ceased, without any bougie.

The following case forms a useful contrast:

*Case* 3. A married man, of unimpeachable morals, and never exposed to venereal infection, became affected at first with a gleety, but afterwards with a puriloid discharge, unattended by any pain or difficulty in making water. A bougie was employed; under the use of which, the irritation increased, the discharge became greater, and his general health suffered. A swelling now formed under the urethra, within the scrotum; which, after much local and constitutional irritation, discharged itself, and the urine passed through the aperture in the scrotum. The bougie was again employed, to extend the urethra and to heal the opening from it into the scrotum; but soon another abscess formed in perineo, and from this the urine became extravasated into the cellular substance about the perineum, and in the scrotum, of which he died. On dissection two ulcers were found, but no stricture. "If this disease (says Mr. Cooper) had been from the first constitutionally treated, instead of being irritated by the injudicious use of bougies, the person would probably have had his life preserved." 203.

Apertures, from loss of substance in the urethra, are generally seated before the scrotum, and are very difficult to heal. They are sometimes from half an inch to an inch in extent. The patient's mind is very uneasy under this affliction, as he considers himself in a great measure emasculated. Mr. Cooper thinks, that the cause of the aperture is an abscess in one of the lacunæ, "with a disposition to the sloughing process; and when the matter is discharged, the slough which follows, removes the lower portion of the urethra opposite to the lacunæ, and thus produces a large aperture." Such cases are generally abandoned by surgeons as incurable; but from some examples here adduced, our author hopes they will not, in future, be viewed in such gloomy colours.

*Case* 4. A gentleman had a chancre at the orifice of the urethra, attended with great inflammation, and ending in sloughing of the canal at the junction of the scrotum and penis, leaving a large fistulous opening, without any disposition to close. The introduction of bougies had no good effect; and neither had the application of blisters, nor the paring of the edges of the fistula, and bringing them together by the twisted suture. Seeing that the scrotum formed two-thirds of the opening, and the skin of the penis one-third, Mr. Cooper conceived the possibility of healing it on the principle of the contraction of the skin in cicatrization. Accordingly, in June 1818, he applied the nitrous acid upon the edge of the fistulous orifice, to the extent of three quarters of an inch around it. The skin sloughed superficially, formed granulations, and healed. It soon afterwards began to contract, so as to show that the principle would ultimately much diminish the orifice. In the succeeding October, the acid was again applied, and with increased effect. In November, the process was repeated by the patient himself, and the opening reduced from the size of a pea to that of a pin. In two applications more the fistula was completely cured. The next instance is still more interesting, and is drawn up by Mr. Hunter, jun. of Tower Street, of whom Mr. Cooper speaks in terms of great eulogy.

CASE 5.—*Abscess in the Urethra.* Mr. H——, ætat. 56, in consequence of violent inflammation in the penis and scrotum, the effect of neglected stricture, had a large abscess opposite the bulb of the urethra, from which was discharged a quantity of fœtid matter. By Mr. Cooper's advice, a silver catheter was introduced, with great difficulty, on account of two firm strictures, and the highly inflamed state of parts. This was worn for three weeks, when another abscess opened at the under part of the urethra, immediately anteriorly to the scrotum. The fistulous orifice of this abscess enlarged to half an inch in length, and all attempts to close it were fruitless. Mr. Cooper performed the following operation, on Taliacotian principles, now so frequently applied to various species of dismemberment.

"December 9th, 1818. An elastic catheter being passed into the bladder, the callous edges of the opening were pared off, so as to produce an entire new surface; a portion of integument was then dissected from the scrotum, (leaving it attached at the upper part) and turned over upon the wound, to which it was exactly fitted; this was held down by four sutures covered by small slips of adhesive plaster; a bandage was applied to support the scrotum, and the patient placed on his back in bed." 209.

On the 15th of December the dressings were removed; the edges of the flap appeared in perfect apposition with the parts beneath, though the integuments were œdematous.

On the 19th, the catheter was withdrawn, being stopped up, and another introduced. From the 15th to the 31st of January, the catheter was withdrawn three or four hours daily; and although some urine occasionally passed by the original fistulous opening, yet, on the 1st of February, the water flowed along the urethra, for the first time, while the catheter was out, and the cure was finally complete. "The penis, (says Mr. Hunter) is somewhat drawn down by the contraction of the integument, and the small pouch which was formed by the ligatures at the upper part of the flap is removed. I have the greatest expectation that this operation will, in others, be found useful, as this gentleman's wound has remained perfectly well for seven months." 212.

It is needless to remark, that this operation does great credit to Mr. Cooper's ingenuity in proposing it, and to Messrs. Hunters' assiduity, skill, and perseverance in bringing it to a successful issue.

II. *On Encysted Tumours.* The species of encysted tumours to which Mr. Cooper confines his attention in this Essay, is that which is situated just under the skin, and so frequently seen upon the head, face, and back; though occasionally under the skin upon other parts of the body. It is nearly globular in figure, and, when seated on the head, feels very firm; while on the face, it gives an obscure sense of fluctuation. The superincumbent skin is uninflamed, though sometimes streaked with blood-vessels of larger calibre than in the neighbouring parts. A dark-coloured spot may often be seen on the skin over the centre of the tumour, which sometimes continues through the whole course of the disease.

The tumour is usually unattended with pain, and is never, in itself, dangerous. It requires removal only from the unsightly appearance which it produces. In a patient of Mr. Hall's, of Dulwich, Mr. Cooper saw sixteen upon the head, some of which, as large as a walnut, he removed. The largest size he has ever met with, was that of a cocoa-nut, on the head. It sprung from the vertex and gave the patient a most grottesque appearance. It was successfully removed by our author. Mr. Cooper thinks these tumours, in some degree, hereditary. When opened, a curd-like substance is generally discharged from them, having a sour, and sometimes an abominable smell. When they acquire any size, there appears to be an attempt at their removal made by Nature. The skin inflames over them; the swelling then becomes painful; ulceration slowly follows, and the curdly substance, mixed with pus, is discharged. The opening sometimes closes; but often remains fistulous, with some inconvenience to the patient. On dissection, they are found to adhere generally, but not universally to the skin. The cyst is found of different thicknesses, according to the site of the tumour. On the head it acquires the greatest density; so much so, as to maintain its form when its contents are discharged. Within the cyst there is a lining of cuticle which adheres to its interior, and several desquamations of the same substance are formed within the first lining, apparently secreted, at different periods of the growth of the cyst. The contained substance bears, to the eye, the character of coagulated albumen; but as it varies much, these swellings got the ridiculou appellations of atheroma, or meliceris. From these cysts horny excrescences sometimes grow, of which a plate is given in the work, representing a curious production of this kind extirpated by Dr. Reots, of Kingston; an account of which may be seen in Rees's Cyclopœdia, under the word, "*Horny excrescence.*" "The manner in which these horny excrescences grow, (says Mr. Cooper) is as follows:—The horn begins to grow from the open surface of the cyst; at first it is soft, but soon acquires considerable hardness; at first it is pliant, but after a few weeks, it assumes the character of horn." "In their removal it is necessary, to prevent their recurrence, that the cyst as well as the horn should be dissected out."

Our author thinks, that the cysts originate from an extremely enlarged follicle, obstructed at its orifice, and thereby incapable of discharging its contents. These follicles appear, on superficial examination, to be merely pores in the skin; but, upon the introduction of a fine probe, they are found to penetrate into the subcutaneneous tissues. They are lined by cuticle, which secretes a sebaceous matter that lubricates and defends the surface of the skin wherever they are found. This matter may be pressed from the follicles of the nose, in the form of worms, very considerably longer than the skin is thick; proving that these pores extend below the cutaneous expansion.

"These encysted tumours begin as follows:—A follicle becomes obstructed at its termination upon the skin. The secretion still proceeding, its sides become extended in the cellular membrane, where it can most easily yield; and this obstruction of its secretion produces a swelling of greater or less magnitude, according to the degree of obstruction and the duration of the disease. If it be said, how is it possible it can be thus extended? the answer is this, other membranes expand to much greater comparative magnitude. An ovarium, which would not contain within its membrane more than two drachms of water, will expand to a magnitude capable of containing ninety-seven pints, for of such an ovarium I have a preparation." 222.

Pressure, Mr. C. thinks, is often the cause of these swellings, as is seen upon the shoulders, where the braces produce them. "But in a diseased state of the secretions, a want of due moisture will produce the same effect, by inspissation of the substance secreted, and by its incapacity to pass the orifice of the follicle." We have often, indeed, considered this as the cause of various cutaneous affections; and the decoctions of the woods and other diluting drinks owe, probably, much of their virtues to their increasing and attenuating the insensible perspiration. The reason why these cysts do not inflame, when opened, Mr. Cooper thinks is, that their internal secreting surface is, in fact, an extension of the cuticle itself.

*Treatment.* If the follicle appears only as a black speck, filled by hardened sebaceous matter, a probe may be passed through it, and the contents squeezed from the tumour. If this cannot be done without violence, which would induce inflammation, the opening should be made larger, and the sebaceous matter evacuated by pressure. For their removal, the common mode is to dissect them out entire. "But the best manner of doing it is not to dissect them out whole, but first to make an incision into them, and then by pressing the sides of the skin together, the cysts may be easily everted and removed. If attempted to be removed whole, the dissection is most tedious, and before it is completed the cyst is either cut or burst. So many incisions, and so much pain may be readily avoided by opening it freely by one incision, and taking it between the forceps to dissect it from its adhesion to the surrounding cellular membrane. When a swelling of this kind in the scalp is to be removed, make an incision from one side of the tumour to the other, directly through its centre; and its contents, which are very solid in this case, are directly discharged in form similar to the tumour; then put a tenaculum into the cyst, raise it, and it becomes most easily separated."

The hair is then to be braided together from each side of the wound, so that the edges may be approximated and retained so; pressure will generally stop any hæmorrhage from small vessels.

The tumour of this description, which takes place at the outer canthus of the eye, is the most difficult to remove. It passes within the orbit, and often adheres to its periosteum. The inner part of the cyst, too, is with great difficulty reached in the operation. The removal of these cysts is not always unattended by danger. Mr. Cooper has seen three instances of severe erysipelatous inflammation on removing these swellings from the scalp, which he believes is owing to the tendon of the occipito-frontalis being wounded, when the tumours were attempted to be dissected out entire.

"Trifling as the aperture appears, occasioned by this operation, care must be taken, for a few days after it, when the swelling is seated on the head. A lady had an encysted tumour on the scalp removed; three days after, she went into a cold bath; soon after she left the bath she was seized with a rigor and severe pain in the head; an erysipelatous inflammation succeeded upon the head and face; and, notwithstanding she had promptly the most able medical assistance in Dr. Baillie, she fell a victim to this inflammation." 225.

We have now concluded our Analytical Review of the two short Essays that close Mr. Cooper's volume. The other Essay, occupying nearly two hundred pages, and containing very interesting communications from a great number of able surgeons, particularly respecting compound dislocations of the ancle-joint, we must reserve for a long article in our succeeding Number. Mr. Cooper appears to be so conscious of the intrinsic value of his *matter*, that he pays little or no attention to the language or *manner* in which it is conveyed. But, in truth, we wonder how he has contrived to find leisure, either for taking the orginal memoranda, or subsequently arranging them for the press. Mr. Cooper, we should presume, is one of the happiest men on earth. He never passes a day without imbruing his hands in gore; and, in all probability, sheds, personally, more human blood than Caligula of old—and that, too, without the least remorse of conscience! The Italian tyrant, wished that Rome had but one head, in order that he might enjoy the pleasure of striking it off. If all the *condemned* members of the British people were to be amputated by a single stroke, Mr. Astley Cooper would be the man to wield the knife. But the parallel will not hold much further, without running by antithesis. Caligula, by his cruelty, destroyed hundreds of his subjects; Cooper, by his skill, has preserved thousands of his countrymen. The *former* was abhorred, and ultimately conspired against, even by the minions of his household, and the instruments of his tyranny; the *latter* is admired, respected, and materially supported by men of science, who are personally unknown to him, and who never did, and never can, receive any favour from his hands!

After all this, will it be believed that a Caligula had friends, and that a Cooper has enemies? Alas! it requires but little penetration to detect the secret springs which prompt to the most discordant actions and sentiments in the drama of human Life! Happy is the man who, with philosophic independence, can view the conflicting interests of the living, wih the same feelings as he peruses the records of the dead! Bad as the world is, however, there are innumerable proofs, and our author affords one of them, that talents, and zeal, and integrity in the medical profession, are quickly recognized by the majority, and ultimately rewarded both in "airy fame" and substantial emolument.

### Art. 13. *Luxation of the Shoulder-Joint.*

[ Kirby's Cases in Surgery, Octavo, 1819. ]

From among the many highly interesting observations with which this little volume abounds, (which we strongly recommend to the surgical reader) we select the SHOULDER-JOINT LUXATION, an accident of very frequent occurrence, with a wood engraving of the process for reduction.

"I provide myself, (says Mr. Kirby) with a piece of strong coarse linen, a yard in breadth, and three yards in length. Along its centre, I make a slit, sufficiently large to admit the luxated member, which is passed through it up to the shoulder-joint. Each end is then twisted two or three times or oftener, until the superior division of the band bears upon the acromion process, and its inferior part acts upon the remainder of the scapula, by which it becomes fixed, together with the trunk. The ends of the bandage are next given into the hands of an assistant, or secured at the opposite side of the neck by a loose noose, until the proper occasion for using them. The ligature which Mr. Hey has described, is, in the next place, applied above the condyles of the humerus, care being taken that the skin forms no plait or fold, and that it is first covered over with some shamoy leather, for protection from excoriation."

The patient now sits upon a mattress stretched upon the floor, and the assistants, to whose management the extension and counter-extension are consigned, place themselves at his sides, sitting opposite to one another. Each disposes a leg under the hams of the patient, until the soles of the feet meet together, while the other legs are stretched towards each other, until they meet, in the same manner, behind him—thus —

If occasion should require a greater power, the assistants may be increased at pleasure; and are to be placed behind the others, with their faces turned to the patient, and their limbs extended at each side of the assistant, at whose back they are placed. The extension is now made, with the arm raised nearly to a right angle with the body, and in a direction forward or backward, as the circumstances of the case seem to require. This is persevered in until it is perceived that the head of the bone, which can be easily felt, and should be pressed upon during the operation, has moved from the new situation which it occupied. Before this change in its position, every attempt to jerk it into its proper articulation, not only disappoints the practitioner, but adds to the future difficulties of the case, by the contusions which must be the consequence, and the violent reaction of the muscles which it excites. When the head of the bone is found to shift its place, the assistants slowly relaxing their force, it is directed towards the glenoid cavity with one hand, while the other, by which the arm is grasped, gently lays the elbow towards the side." 56, 57.

Mr. Kirby admonishes us not to despair, in cases of long duration, even although the first efforts have failed. He has persevered for hours, after every hope of success had deserted his assistants, and succeeded in the end. This subject is illustrated by many interesting cases and highly judicious observations, which we particularly recommend to our chirurgical brethren.

## Figures and Tables

**Figure f1:**